# Impact of diabetes on COVID‐19 mortality and hospital outcomes from a global perspective: An umbrella systematic review and meta‐analysis

**DOI:** 10.1002/edm2.338

**Published:** 2022-04-20

**Authors:** Stavroula Kastora, Manisha Patel, Ben Carter, Mirela Delibegovic, Phyo Kyaw Myint

**Affiliations:** ^1^ School of Medicine, Medical Sciences & Nutrition University of Aberdeen Aberdeen UK; ^2^ Department of Biostatistics and Health Informatics Institute of Psychiatry, Psychology & Neuroscience, King's College London London UK; ^3^ Aberdeen Cardiovascular and Diabetes Centre (ACDC) Institute of Medical Sciences (IMS), University of Aberdeen Aberdeen UK; ^4^ Aberdeen Cardiovascular and Diabetes Centre (ACDC) Institute of Applied Health Sciences, University of Aberdeen Aberdeen UK

**Keywords:** COVID‐19, diabetes, discharge, disease severity, intensive care, mortality, ventilation

## Abstract

**Introduction:**

To date, COVID‐19 has claimed 4.9 million lives. Diabetes has been identified as an independent risk factor of serious outcomes in people with COVID‐19 infection. Whether that holds true across world regions uniformly has not been previously assessed.

**Methods:**

This study offers the first umbrella systematic review and meta‐analysis to analyse the collective and geographically stratified mortality, ICU admission, ventilation requirement, illness severity and discharge rate amongst patients with diabetes. Five databases (EMBASE, MEDLINE, CAB Abstracts, PsychInfo and Web of Science) and 3 additional sources (SSRN's eLibrary, Research Square and MedRxiv) were searched from inception to 30 August 2021. Prospective and retrospective cohort studies, reporting the association between diabetes and one or more COVID‐19 hospitalization outcomes, were included. This meta‐analysis was registered on PROSPERO, CRD42021278579. Abbreviated MeSH terms used for search were as follows: (Diabetes) AND (2019 Novel Coronavirus Disease), adapted per database requirements. Exclusion criteria exclusion criteria were as follows: (1) none of the primary or secondary outcomes of meta‐analysis reported, (2) no confirmed COVID‐19 infection (laboratory or clinical) and (3) no unexposed population (solely patients with diabetes included). Quality of the included studies were assessed using the Newcastle‐Ottawa Scale (NOS) whilst quality of evidence by the GRADE framework. Studies that were clinically homogeneous were pooled. Summative data and heterogeneity were generated by the Cochrane platform RevMan (V. 5.4).

**Results:**

Overall, 158 observational studies were included, with a total of 270,212 of participants, median age 59 [53–65 IQR] of who 56.5% were male. A total of 22 studies originated from EU, 90 from Far East, 16 from Middle East and 30 from America. Data were synthesized with mixed heterogeneity across outcomes. Pooled results highlighted those patients with diabetes were at a higher risk of COVID‐19‐related mortality, OR 1.87 [95%CI 1.61, 2.17]. ICU admissions increased across all studies for patients with diabetes, OR 1.59 [95%CI 1.15, 2.18], a result that was mainly skewed by Far East‐originating studies, OR 1.94 [95%CI 1.51, 2.49]. Ventilation requirements were also increased amongst patients with diabetes worldwide, OR 1.44 [95%CI 1.20, 1.73] as well as their presentation with severe or critical condition, OR 2.88 [95%CI 2.29, 3.63]. HbA1C levels under <70 mmol and metformin use constituted protective factors in view of COVID‐19 mortality, whilst the inverse was true for concurrent insulin use.

**Conclusions:**

Whilst diabetes constitutes a poor prognosticator for various COVID‐19 infection outcomes, variability across world regions is significant and may skew overall trends.

## Graphical abstract

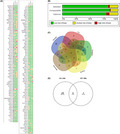

## 1 INTRODUCTION

COVID‐19, a novel coronavirus identified in late 2019, has rapidly spread worldwide resulting in the first pandemic experienced in the modern world since 1918.^1^ Currently, more than 220 million have been infected, with 4.9 million deaths as of 18 October 2021. Metabolic conditions, and primarily diabetes, have emerged since the beginning of the pandemic as significant risk factors for poor COVID‐19 outcomes.^2^ A wealth of observational studies and consequently meta‐analyses have attempted to quantify the association of diabetes as an independent risk factor of poor COVID‐19 outcomes and consistently found that diabetes is associated with poorer outcomes across this patient group.

Until present and to the best of our knowledge, an umbrella systematic review and meta‐analysis has not been conducted to collectively assess available meta‐analyses. Furthermore, whilst patient ethnicity as well as global discrepancies of healthcare facilities and antidiabetic medication access are well‐established variables,^3^, ^4^, ^5^, ^6^, ^7^ no previous work has factored in, study geographical origin to assess the potential impact of these parameters on COVID‐19 outcomes in patients with diabetes.

We primarily aim to quantify the overall impact of diabetes in COVID‐19 across three main outcomes: mortality, ICU admission and ventilation (invasive and non‐invasive). Secondary outcomes include illness severity, discharge rate, identification of putative geographical variability across outcomes and associated factors of poorer or improved prognosis, amongst patients with diabetes.

## 2 METHODS

### 2.1 Search strategy and selection criteria

**FIGURE 1:**
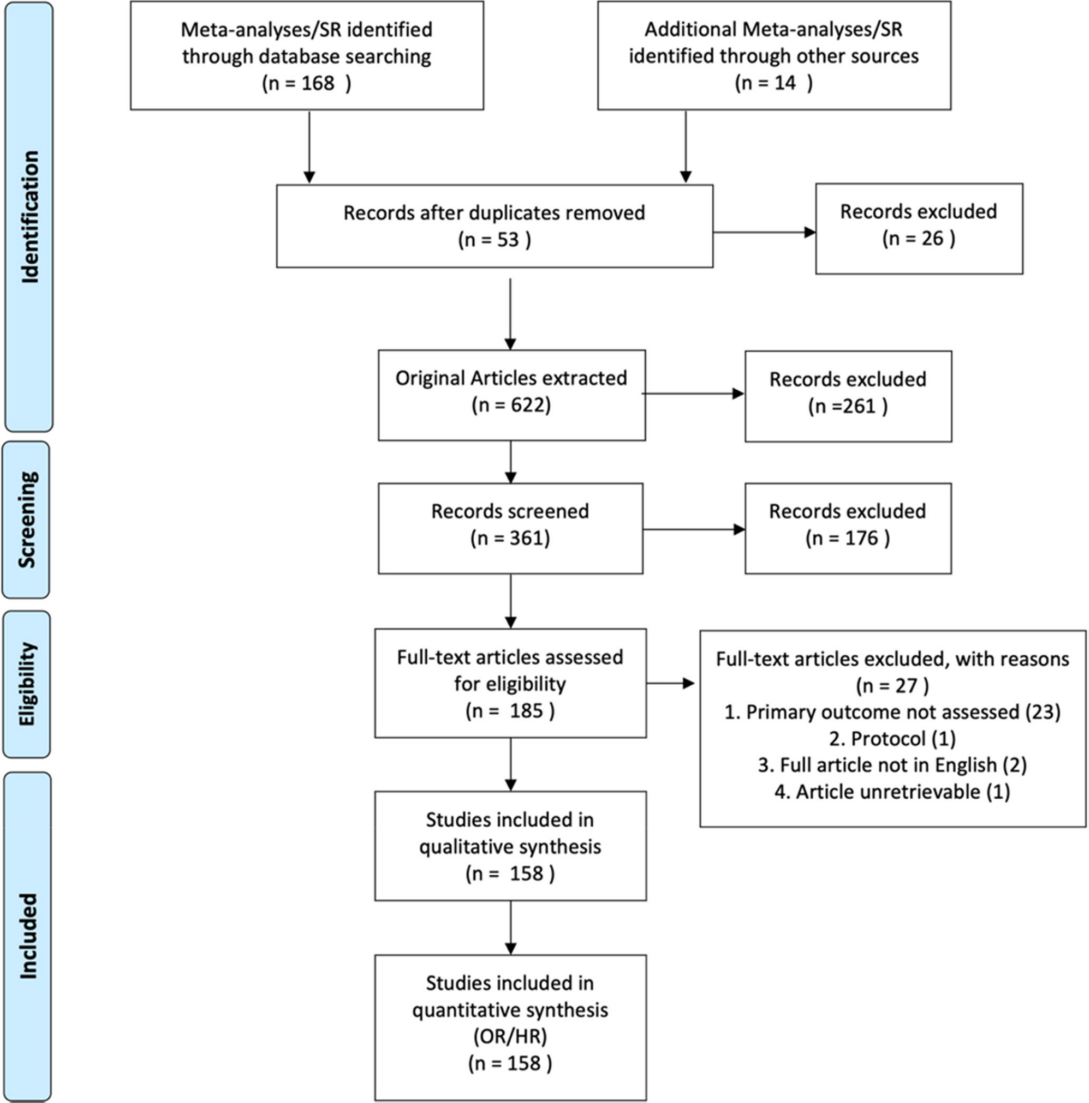
PRISMA 2009 flow diagram. Search strategy included and excluded studies^179^

A systematic literature review was conducted according to the guidelines of the Preferred Reporting Items for Systematic Reviews and Meta‐Analyses (PRISMA) (Figure 1). For the present study, a protocol was prospectively registered at the PROSPERO database (CRD42021278579), amended on the 14 October 2020 to extend date of expected submission. Independent literature search for relevant studies, restricted to systematic reviews and meta‐analyses, was performed up to 30 August 2021 on five databases: EMBASE, MEDLINE, CAB Abstracts, PsychInfo and Web of Science. Additional records were identified through other sources, including SSRN's eLibrary, Research Square and MedRxiv to reduce publication bias. The MedRxiv search was simplified according to database search functionality. The references of the included systematic review and meta‐analysis studies were scrutinized for additional relevant studies.

The following search term was used in OVID: (Diabetes OR T2DM OR T1DM OR Diabetes mellitus).mh,tw,ab,hw,kw. AND (2019 Novel Coronavirus Disease OR 2019 Novel Coronavirus Infection OR 2019‐nCoV Disease OR 2019‐nCoV Infection OR COVID‐19 Pandemic OR COVID‐19 Pandemics OR COVID‐19 Virus Disease OR COVID‐19 Virus Infection OR COVID19 OR Coronavirus Disease 2019 OR Coronavirus Disease‐19 OR SARS Coronavirus 2 Infection OR SARS‐CoV‐2 Infection).mp. limit to (English language and humans). The same search strategy was adapted for the remaining databases.

**FIGURE 2:**
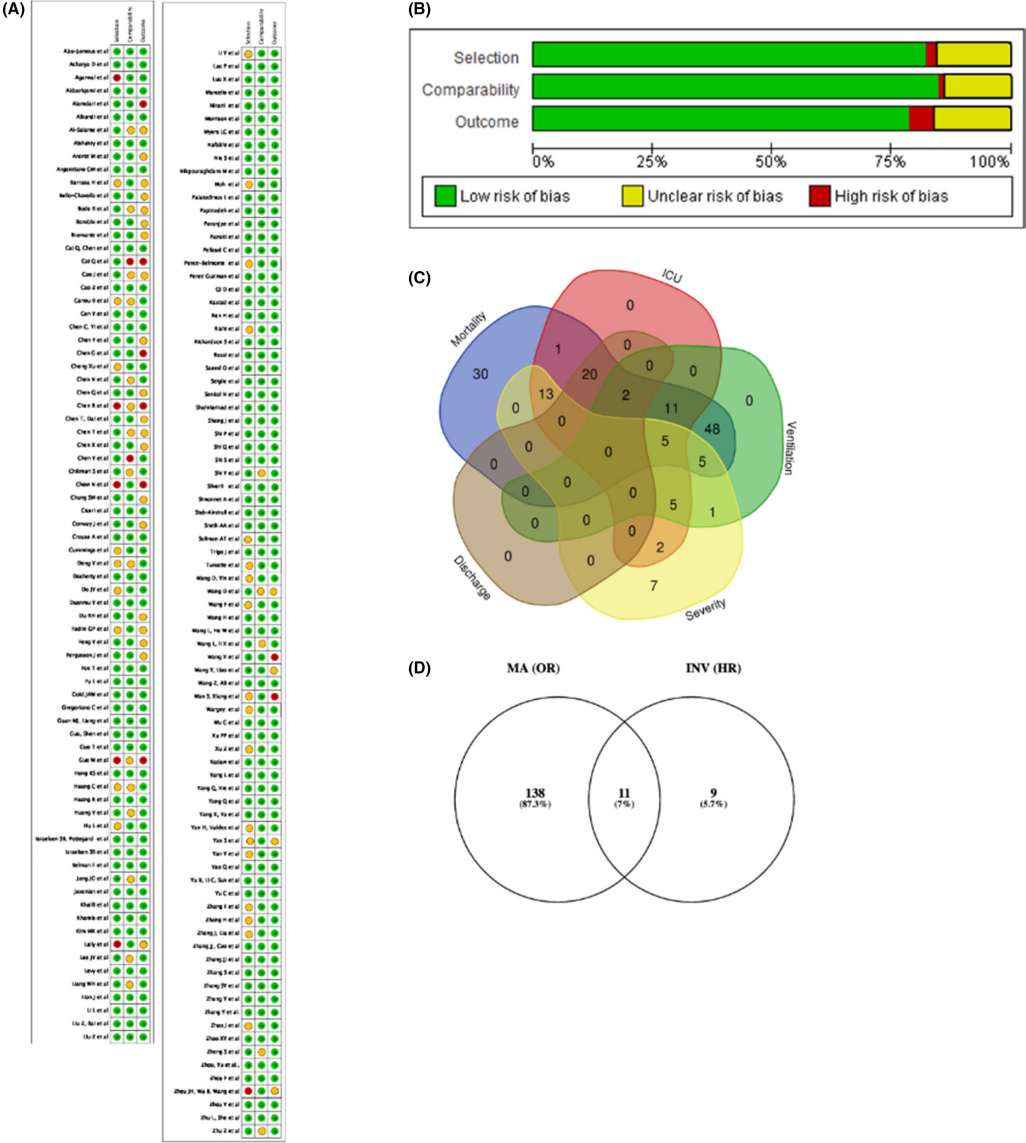
Risk of bias graphs and study data extraction strategy. (A) Review authors' judgements about each risk of bias item per included study. Review authors' judgements about each risk of bias item presented as percentages across all included studies (B). Outcomes addressed by total number of studies and overlap (C), Number of studies used for addressing primary and secondary outcomes (D)

Prospective and retrospective cohort studies were extracted from eligible systematic reviews and meta‐analyses to enable umbrella systematic review of available data as described in Aromataris et al.,^8^ examining COVID‐19 mortality, ICU admission, ventilation requirement, disease severity and discharge in the context of diabetes (Table S1). Restrictions included English language and human. After removing duplicates (EndNote V.20), citations were screened by title and abstract; then, full texts were appraised to determine their eligibility by two authors (SK and MP) (Figure 2). Two authors (SK and MP) independently conducted the abstract and full‐text screening. Disagreements were resolved by a consensus meeting. Peer‐reviewed full‐text papers that reported one or more of the primary outcomes were selected. Full‐text exclusion criteria were as follows: (1) none of the primary or secondary outcomes of meta‐analysis reported, (2) no confirmed COVID‐19 infection (laboratory or clinical) and (3) no unexposed population (solely patients with diabetes included). Excluded studies and justifications are recorded in Table S1. Data from each article was extracted by two authors (SK and MP) and validated independently by author crossover: (1) Total number of participants, type of study, setting of study (hospital/community), sample size (total), patients with diabetes (total), Number of patients with T1DM or T2DM if available [*N*; %], mortality [*N*; %], ICU admission [*N*; %], Severity (mild, moderate, severe/critical [*N*;%], ventilation required included both non‐invasive [Continuous positive airway pressure (CPAP) Biphasic Positive Airway Pressure (BiPAP), High‐Flow Nasal Cannula (HFNC) and invasive ventilation application, positive end‐expiratory pressure (PEEP)] events [*N*; %], discharge rate [*N*; %], patient characteristics: age, gender method of COVID‐19 diagnosis.

Quality of the included studies were assessed by two independent reviewers (SK and MP) using the Newcastle‐Ottawa Scale (NOS) for observational studies.^9^ Studies were of high quality if a NOS score ≥6 was achieved.^10^ Adequate follow‐up was ≥30 days (Table S1). Overall grading the quality of evidence was assessed by the GRADE framework.^11^ Heterogeneity was assessed using *I* ^2^.

### 2.2 Study outcomes

Study primary outcomes included mortality, ICU admission and ventilation requirement events. These were defined as the proportion of people with an event, of each respective outcome, in comparison to people without the event, in the same population. Secondary outcomes were disease severity [mild, moderate and severe/critical] (events) and discharge events amongst patients with diabetes vs. without. Stratified analysis was conducted by global geographical region to identify sources of heterogeneity amongst world regions.

Confounding factors of increased mortality were assessed using generic inverse variance model regression (IVR), adjusted with covariates consistent with the primary outcome and expressed as random effect (RE), hazard ratio (HR) measures. Variables assessed included age (continuous variable), gender (categorical variable), smoking status (categorical), alcohol misuse (categorical), HbA1C ≤ 70 mmol vs. >70 mmol (categorical), diabetes type (Type 1 vs. Type 2) (categorical), insulin use (categorical), metformin use (categorical), DPP4 inhibitor use (categorical), cardiovascular comorbidities including myocardial infarction, ischaemic cardiomyopathy, hypertension (categorical), acute and chronic kidney injury (categorical), immunocompromised (categorical), biochemical findings (including white blood count, C‐reactive protein) (continuous). Both crude (unadjusted) and adjusted HRs were presented with associated 95% confidence intervals (CI) (Figure S3). For crude HRs, antidiabetic medication brand, dose and duration of action were not possible to factor in, due to lack of data reporting in individual studies. Adjusted HR (95% CI) of mortality amongst patients with diabetes was adjusted for age, gender, cardiovascular comorbidities, biochemical findings, smoking/alcohol use, immunocompromised status and medications (Figure S4).

Mortality was measured as 28‐ or 30‐day death events or till the end of follow‐up of each individual study (Table S1). Illness severity was assessed by CURB‐65 stratification score; Guidance for Corona Virus Disease 2019 (6th edition) released by the National Health Commission of China,^12^, ^13^ modified version of the WHO/International Severe Acute Respiratory and Emerging infection Consortium case record form for severe acute respiratory infections,^12,13^ or the necessity for the use of a high‐flow nasal cannula, mechanical ventilation, CRRT, or ECMO, or admission to an ICU, of as a respiratory rate > 30/min, oxygen saturation ≤ 93%, PaO2/FiO2 ≤ 300 mm Hg., with shock or respiratory failure, mechanical ventilation requirement, or combined with other organ failure, requiring admission to intensive care unit (ICU). Individual severity definition per study is presented in Table S1.

### 2.3 Data analysis

Clinical context and design were compared and where appraised as homogeneous, studies were considered as suitable for pooling.^14^ The meta‐analysis was conducted by computing the pooled odds ratio (OR) as per Haensel–Mantel model or Hazard ratio (HR) as per inverse variance analysis, random effects (RE) with Review Manager (RevMan) V 5.4 software. Statistical heterogeneity was quantified using *I* ^2^ statistics and Cochrane *Q* tests.

#### 2.3.1 Assessment of heterogeneity and subgroups to explain differences

Only studies that are clinically homogeneous were pooled. Heterogeneity was assessed using *I* ^2^, and *I* ^2^ greater than 70% was explored using subgroups.^14^ The following subgroups were used to explain the heterogeneity: risk of bias; age, geography, study design (prospective). Asymmetry was assessed by funnel plot, and asymmetry was assessed formally by rank correlation test (Begg's test; RevMan V. 5.4). Sensitivity analyses were conducted to assess the impact of individual potential confounding variables. Publication bias was assessed visually by funnel plot, and asymmetry was formally assessed, by rank correlation test (Begg's test).^15^

## 3 RESULTS

Following the PRISMA guidelines on systematic review search, we identified 53 eligible meta‐analyses studies for study extraction. Post‐individual study extraction and duplicate study removal, we identified 185 studies eligible for full‐text screening (Figure 2). Full‐text screening excluded 27 studies (Table S1). A total of 158 studies remained,^16^, ^17^, ^18^, ^19^, ^20^, ^21^, ^22^, ^23^, ^24^, ^25^, ^26^, ^27^, ^28^, ^29^, ^30^, ^31^, ^32^, ^33^, ^34^, ^35^, ^36^, ^37^, ^38^, ^39^, ^40^, ^41^, ^42^, ^43^, ^44^, ^45^, ^46^, ^47^, ^48^, ^49^, ^50^, ^51^, ^52^, ^53^, ^54^, ^55^, ^56^, ^57^, ^58^, ^59^, ^60^, ^61^, ^62^, ^63^, ^64^, ^65^, ^66^, ^67^, ^68^, ^69^, ^70^, ^71^, ^72^, ^73^, ^74^, ^75^, ^76^, ^77^, ^78^, ^79^, ^80^, ^81^, ^82^, ^83^, ^84^, ^85^, ^86^, ^87^, ^88^, ^89^, ^90^, ^91^, ^92^, ^93^, ^94^, ^95^, ^96^, ^97^, ^98^, ^99^, ^100^, ^101^, ^102^, ^103^, ^104^, ^105^, ^106^, ^107^, ^108^, ^109^, ^110^, ^111^, ^112^, ^113^, ^114^, ^115^, ^116^, ^117^, ^118^, ^119^, ^120^, ^121^, ^122^, ^123^, ^124^, ^125^, ^126^, ^127^, ^128^, ^129^, ^130^, ^131^, ^132^, ^133^, ^134^, ^135^, ^136^, ^137^, ^138^, ^139^, ^140^, ^141^, ^142^, ^143^, ^144^, ^145^, ^146^, ^147^, ^148^, ^149^, ^150^, ^151^, ^152^, ^153^, ^154^, ^155^, ^156^, ^157^, ^158^, ^159^, ^160^, ^161^, ^162^, ^163^, ^164^, ^165^, ^166^, ^167^, ^168^, ^169^, ^170^, ^171^, ^172^ all of which were included in the systematic review and 149 were included in the meta‐analysis (Figures 1 and 2; Figure S2, Table S1).^16^, ^17^, ^18^, ^19^, ^20^, ^21^, ^22^, ^23^, ^24^, ^25^, ^26^, ^27^, ^28^, ^29^, ^30^, ^31^, ^32^, ^33^, ^34^, ^35^, ^36^, ^37^, ^38^, ^39^, ^40^, ^41^, ^42^, ^43^, ^44^, ^45^, ^46^, ^47^, ^48^, ^49^, ^50^, ^51^, ^52^, ^53^, ^54^, ^55^, ^56^, ^57^, ^58^, ^59^, ^60^, ^61^, ^62^, ^63^, ^64^, ^65^, ^66^, ^67^, ^68^, ^69^, ^70^, ^71^, ^72^, ^73^, ^74^, ^75^, ^76^, ^77^, ^78^, ^79^, ^80^, ^81^, ^82^, ^83^, ^84^, ^85^, ^86^, ^87^, ^88^, ^89^, ^90^, ^91^, ^92^, ^93^, ^94^, ^95^, ^96^, ^97^, ^98^, ^99^, ^100^, ^101^, ^102^, ^103^, ^104^, ^105^, ^106^, ^107^, ^108^, ^109^, ^110^, ^111^, ^112^, ^113^, ^114^, ^115^, ^116^, ^117^, ^118^, ^119^, ^120^, ^121^, ^122^, ^123^, ^124^, ^125^, ^126^, ^127^, ^128^, ^129^, ^130^, ^131^, ^132^, ^133^, ^134^, ^135^, ^136^, ^137^, ^138^, ^139^, ^140^, ^141^, ^142^, ^143^, ^144^, ^145^, ^146^, ^147^, ^148^, ^149^, ^150^, ^151^, ^152^, ^153^, ^154^, ^155^, ^156^, ^157^, ^158^, ^159^, ^160^, ^161^, ^162^, ^163^

### 3.1 Included study designs

Ten [*N*:10] studies were prospectivewhilst the remaining [*N*:148] retrospective observational (Table S1). A total of fifteen studies were preprints [*N*:15] (Table S1). All studies [*N*:157] included patients from a hospital setting, either ward level care or specialized COVID‐19 wards with the exception of one, which was conducted in a care home setting.^162^ Total patient sample was comprising a total of 270,212 patients, of which 57,801 were diagnosed with diabetes. A total of 488 patients were diagnosed with Type 1 whilst the remaining patients [*N*:57313] with type 2 diabetes.

Median age of total patient sample was 59 [53–65 IQR^25th–75th percentile^] (Figure S1A). Over half (56.5%) [*N*: 105778/187253] of the COVID‐19‐positive patients were male (Figure S1B). Medians were calculated on percentage values to enable comparability across studies. Overall sample mortality was 13.45% (Median) [1.63–25.28 IQR^25th–75th percentile^] across of studies (Figure S1C), ventilation rate at 12.25% (median) [4.16–25 IQR^25th–75th percentile^], ICU admission 18.76% (median) [14.56–37.17 IQR^25th–75th percentile^] and discharge at 67.78% (median) [41.63–88.53 IQR^25th–75th percentile^] at end of study follow‐up, as per individual study (Table S1). Only mortality was found to be significantly different amongst patients with diabetes vs. without diabetes crude numbers (Figure S1C). A total of 22 studies were conducted in EU (Denmark, France, Italy, Spain, Switzerland and United Kingdom), 90 in Far East (China, Korea), 16 in Middle East (Iran, Iraq, Israel Kuweit, Oman, Qatar, Turkey) and 30 in America (29 from the United States and 1 from Mexico).

### 3.2 Risk of bias

We (SLK and MP) employed the NOS for quality assessment.^9^ Ninety‐nine (99) studies were graded as good, forty‐two (42) as fair and eighteen (18) studies as poor according to independent grading as per NOS selection, comparability and outcome parameters (Table S1). Overall quality of evidence was assessed with the GRADE framework and was found to be high (Table S1).^11^

### 3.3 Primary outcomes

#### 3.3.1 Mortality

**FIGURE 3:**
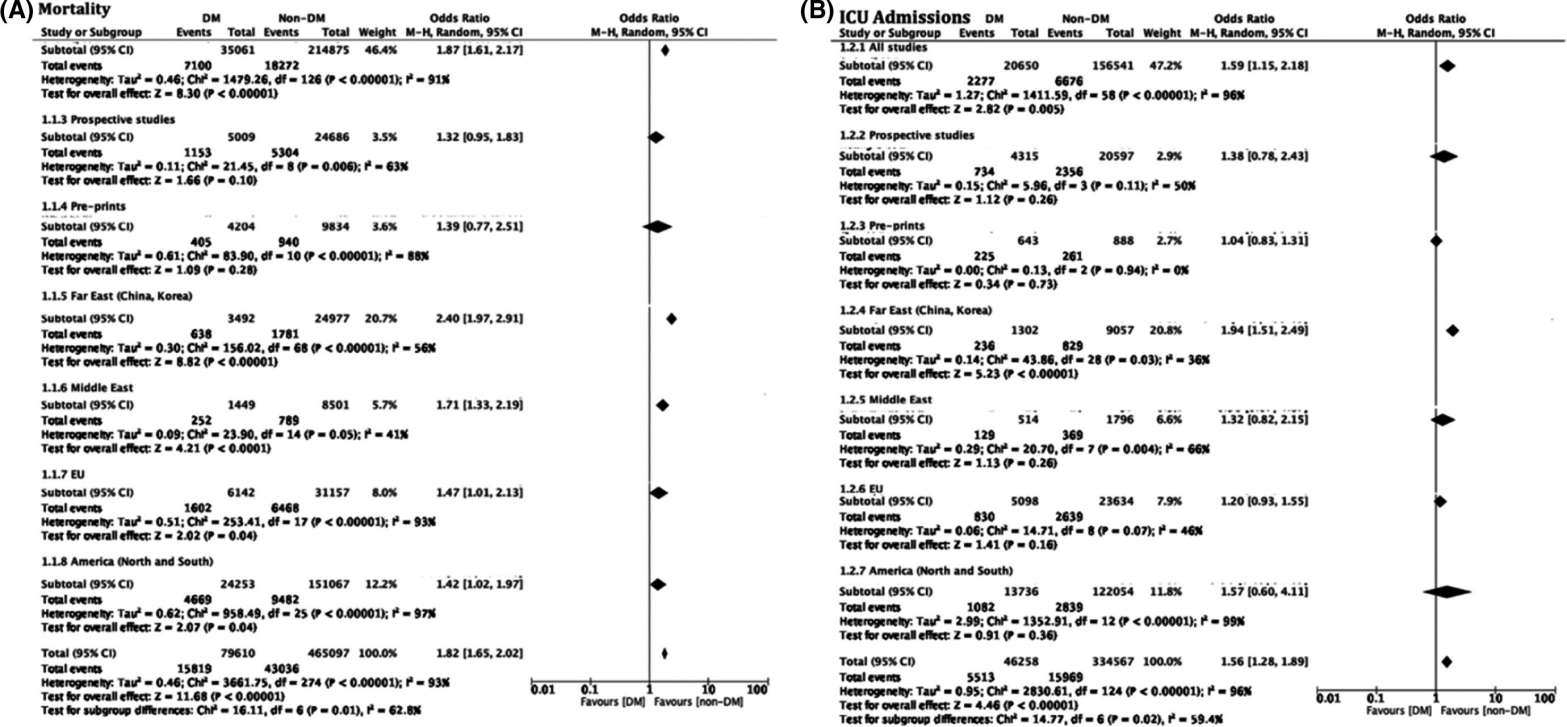
Odds associated with decreased mortality (A) or ICU admission requirement (B). Haensel–Mantel statistical method with odds ratio (random effects) as output only for included observational studies and subgroups as per subgroup title. Summative forest plots of included observational studies of the meta‐analysis (patients with Diabetes vs. without representing respective reduction in mortality (A) or ICU admission (B) rate as per patient population. Forrest and associated funnel plots (Figure S2A,B) were generated with Review Manager V. 5.4 Cochrane Tool for meta‐analysis

A total of 136 studies were included in the analysis of mortality as an outcome (Figure 3A; Figure S2A, Table S1). Overall, studies supported the previously reported increased mortality in patients with diabetes, OR 1.75 [95%CI 1.61, 2.17], *p* < .0001, *I* ^2^ = 91% (Figure 3A; Figure S2A). Heterogeneity was explored and explained by geographical region, with Far East studies (*N*: 77), indicating increased mortality with diabetes OR 2.40 [1.97, 2.91], *I* ^2^ = 56%, Middle East studies (*N*: 15), OR 1.71 [1.33, 2.19], *p* < .0001, *I* ^2^ = 41%, EU studies (*N*: 18), OR 1.47 [1.01, 2.13], *p* = .04, *I* ^2^ = 93% and American studies (*N*: 26), OR 1.42 [1.02, 1.97], *p* = .04, *I* ^2^ = 97% (Figure 3A, Table S1). Overall, mortality amongst the patients with diabetes was found to be higher in the Far East and Middle East world regions. Of note, prospective studies (*N*: 10) did not overall identify a significant increase of mortality amongst two patient groups, OR 1.32 [0.95, 1.83], *p* = .1, *I* ^2^ = 63% (Figure 3A, Table S1). Mortality was explored amongst patients with type 1 vs. type 2 diabetes. Only two studies^82^, ^117^ reported crude numbers of patients with type 1 or type 2 diabetes deaths, suggesting that patients with type 2 diabetes had worse outcomes in respect to mortality, OR 0.68 [95% CI 0.24, 1.87], *I* ^2^ = 0%. albeit the lack of statistical significance, possibly due to the limited sample size (*p* = .45, *N*: 308) (Figure S3D).

#### 3.3.2 ICUadmission

A total of 59 studies were included in the analysis of ICU admission as an outcome (Figure 3B; Table S1). Overall, studies supported the previously reported increased requirement for ICU admission amongst patients with diabetes, OR 1.59 [1.15, 2.18], *p* = .005, *I* ^2^ = 96% (Figure 3B; Figure S2B). Heterogeneity was explored and explained by geographical region, with Far East studies (*N*: 29) indicating increased ICU admission requirement with diabetes, OR 1.94 [1.51, 2.49], *p* < .0001, *I* ^2^ = 36%, Middle East studies (*N*: 8), OR 1.32 [0.82, 2.15], *p* = .26, *I* ^2^ = 66%, EU studies (*N*: 9), OR 1.20 [0.93, 1.55], *p* = .16, *I* ^2^ = 46% and American studies (*N*: 13), OR 1.57 [0.60, 4.11], *p* = .36, *I* ^2^ = 99%. Of note, prospective (*N* = 4), 1.38 [0.78, 2.43], *p* = 0.26, *I* ^2^ = 50%, middle Eastern, European, and American studies did not reach statistical significance for this outcome (Figure 3B).

#### 3.3.3 Ventilation requirement

**FIGURE 4:**
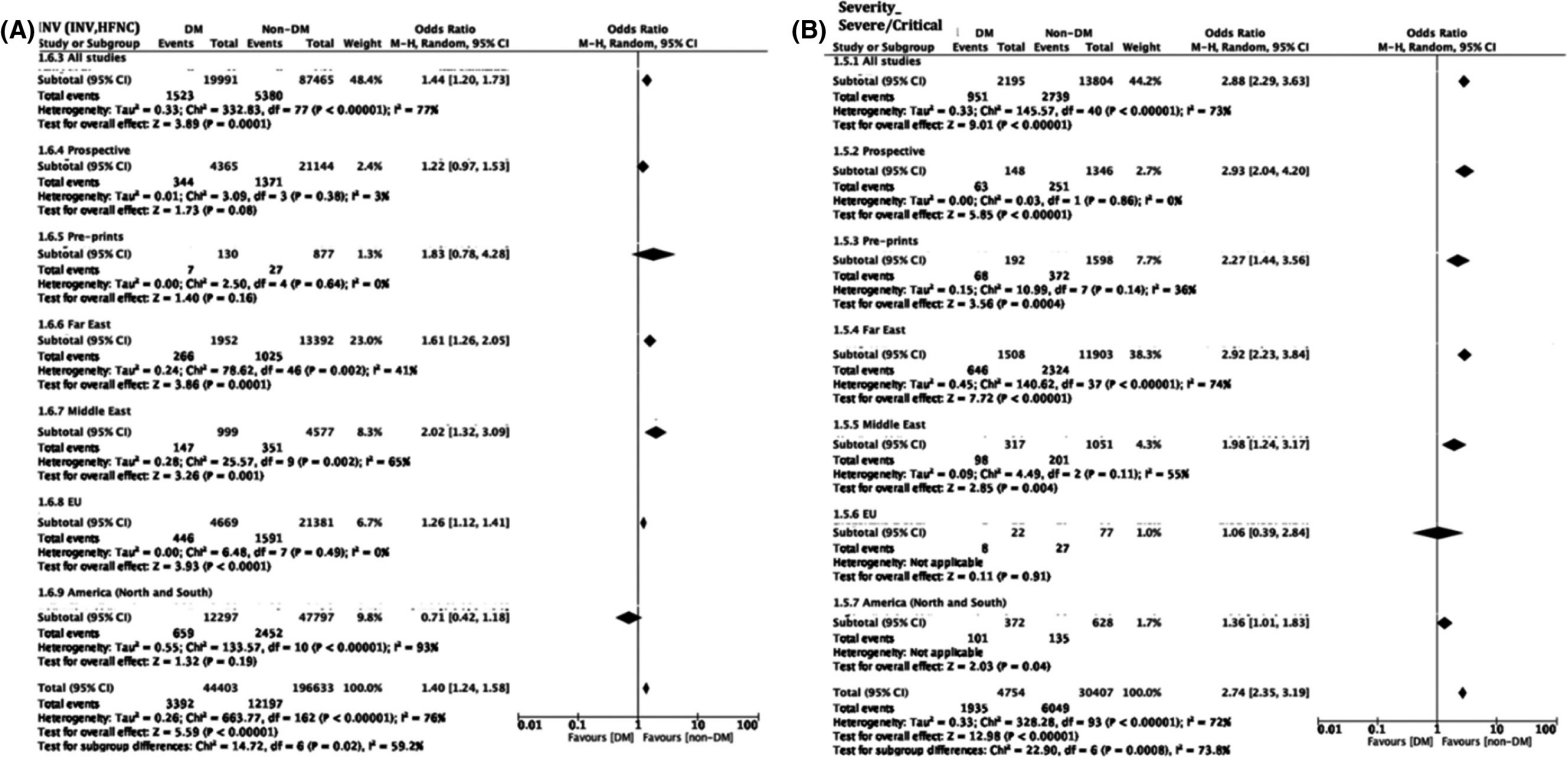
Odds associated with an increased ventilation (invasive and non‐invasive) requirement in patients with diabetes (A) and patients with diabetes presenting with severe or critical condition (B). Haensel–Mantel statistical method with odds ratio (random effects) as output only for included observational studies and subgroups as per subgroup title. Summative forest plots of included observational studies of the meta‐analysis (patients with Diabetes vs. without representing those with increased ventilation requirement (A) or those presenting with severe or critical illness (B) as per patient population. Illness severity definitions per included study are as presented in Table S1. Forrest and associated funnel plots (Figure S2C,D) were generated with Review Manager V. 5.4 Cochrane Tool for meta‐analysis

A total of 83 studies were included in the analysis of ventilation requirement as an outcome amongst patients with diabetes vs. without (Figure 4A, Table S1). Overall, studies supported the previously reported increased requirement for ventilation with diabetes, OR 1.44 [1.20, 1.73], *p* < .0001, *I* ^2^ = 77% (Figure 4A; Figure S2C). Heterogeneity was explored and explained by geographical region, with Far East studies (*N*: 51) indicating increased ventilation requirements with diabetes, OR 1.61 [1.26, 2.05], *p* = .0001, *I* ^2^ = 41%, Middle East studies (*N*: 10), OR 2.02 [1.32, 3.09], *p* = .01, *I* ^2^ = 65%, EU studies (*N*: 8), OR 1.26 [1.12, 1.41], *p* < .0001, *I* ^2^ = 0% and American studies (*N*: 14), OR 0.71 [0.42, 1.18], *p* = .19, *I* ^2^ = 93% (Figure 4A, Table S1). Of note, American studies indicated a decrease of ventilation requirement in patients with diabetes, albeit the lack of statistical significance.

### 3.4 Secondary outcomes

#### 3.4.1 Disease severity

A total of 43 studies were included in the analysis of disease severity (severe or critical) as an outcome amongst patients with diabetes vs. without (Figure 4B; Figure S2D, Table S1). Overall, studies indicated increased patient numbers with diabetes presenting in severe or critical condition, OR 2.88 [2.29, 3.63], *p* < .0001, *I* ^2^ = 73% (Figure 4B; Figure S2D). The reverse trend was observed for patients with diabetes presenting with mild disease severity, OR 0.45 [0.33, 0.61], *p* < .0001, *I* ^2^ = 83% (Figure S5). Heterogeneity was explored and explained by geographical region, with Far East studies (*N*: 38) indicating increased numbers of patients with diabetes presenting with severe condition, OR 2.92 [2.23, 3.84], *p* = .0001, *I* ^2^ = 74% and Middle East studies (*N*: 3),^27^, ^32^, ^105^ OR 1.98 [1.24, 3.17], *p* = .004, *I* ^2^ = 55%. EU^24^ and America^20^ world region subgroupings were not effective given that only two studies reported patients in severe or critical condition for these world regions.

#### 3.4.2 Discharge

**FIGURE 5:**
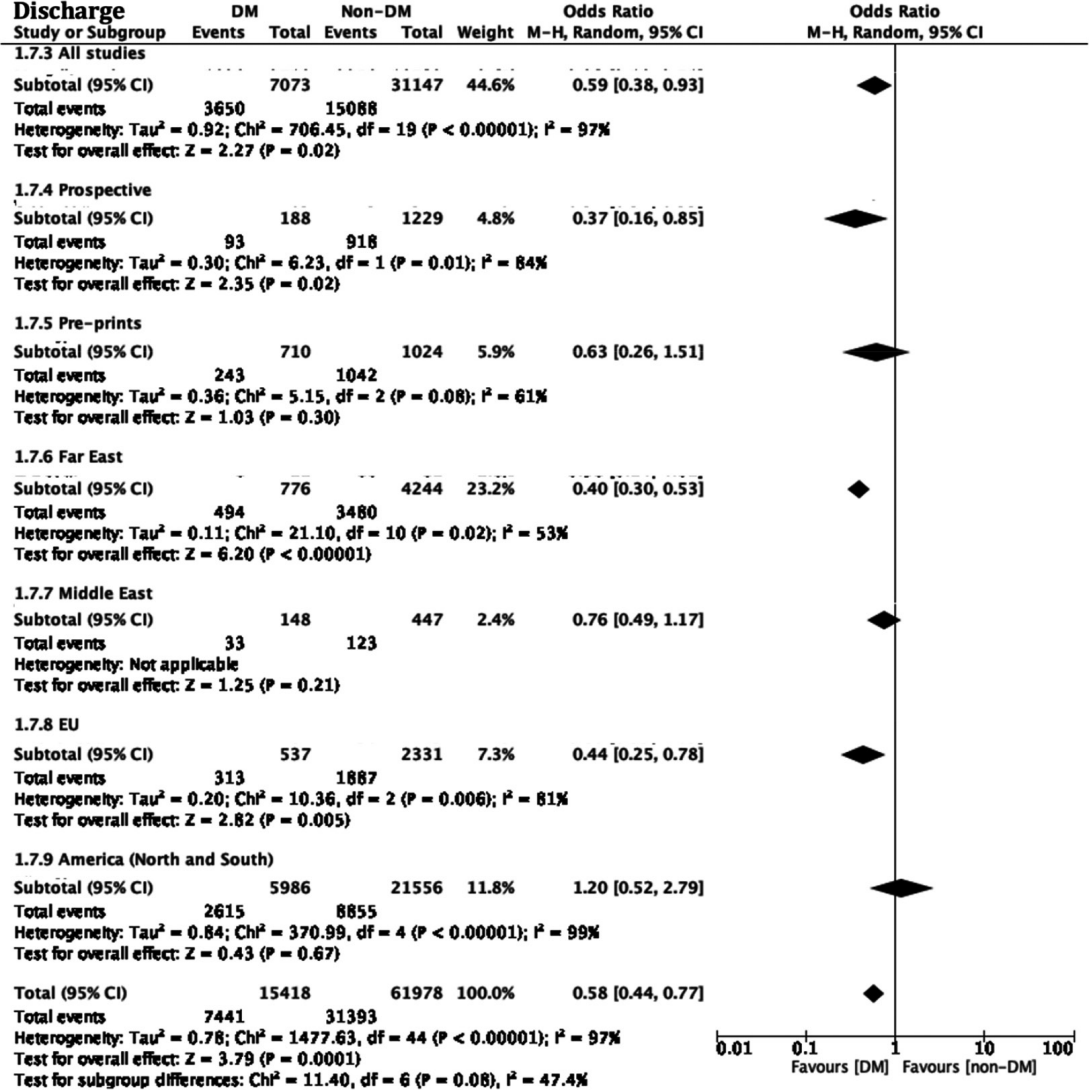
Odds associated with patient discharge at the end‐of study follow‐up. Haensel–Mantel statistical method with odds ratio (random effects) as output only for included observational studies and subgroups as per subgroup title. Summative forest plot of included observational studies of the meta‐analysis (patients with Diabetes vs. without) representing respective discharge odds between the two populations. Forrest and associated funnel plots (Figure S2E) were generated with Review Manager V. 5.4 Cochrane Tool for meta‐analysis

A total of 22 studies reported patient discharge as an outcome amongst patients with diabetes vs. without (Figure 5, Figure S2E, Table S1). Summative results indicated decreased numbers of patients with diabetes being discharged by the end of each individual study follow‐up OR 0.59 [0.38, 0.93], *p* = .02, *I* ^2^ = 97% (Figure 5; Figure S2E). This finding was congruent across world regions, with Far East studies (*N*: 11) OR 0.40 [0.30, 0.53], *p* = .0001, *I* ^2^ = 53%, EU studies (*N*: 3),^110^, ^115^, ^117^ OR 0.44 [0.25, 0.78], *p* = .004, *I* ^2^ = 81% and American studies (*N*: 7), OR 1.20 [0.52, 2.79] *p* = .0001, *I* ^2^ = 99%. Middle East world subgrouping was not feasible for this outcome given that only one study reported this outcome.^105^

#### 3.4.3 Sensitivity analysis

We sought to identify confounding factors that may correlate with COVID‐19 mortality across included studies (Figure S3). Overall, age over 65 years, HR 3.27 [2.83, 3.77], *p* < .0001 (Figure S3A),^58^, ^157^, ^172^ HbA1C over 70 mmol, HR 2.75 [2.60, 2.91] *p* < .0001 (Figure S3C), insulin use HR 2.80 [2.29, 3.44], *p* < .0001 (Figure S3E), were found to increase the risk of mortality amongst patients with diabetes. The use of metformin was associated with decreased risk of mortality, HR 0.60 [0.54, 0.67], *p* < .0001 (Figure S3F)whilst smoking (Figure S3B),^75^, ^166^ diabetes type (Figure S3D)^82^, ^117^ and DPP4 inhibitor use (Figure S3G) were not identified as either risk or protective factors in the context of mortality of patients with diabetes with a COVID‐19 infection. Patients with diabetes had worse outcomes as displayed in the adjusted hazard ratio model, adjusted for age, gender and cardiovascular comorbidities, smoking status, alcohol abuse, immunocompromised, dementia and medications (HR 5.34 [95%CI 2.49, 11.45], *p* < .0001) (Figure S4).

## 4 DISCUSSION

Whilst overall patient mortality has decreased since the beginning of the pandemic, attributable to variable clinical and non‐clinical factors, metabolic conditions, amongst which diabetes, have emerged as significant risk factors for poor COVID‐19 outcomes.^2^

The present work is the first systematic review to assess outcomes of patients with diabetes in the context of COVID‐19 infection whilst accounting for geographical location of outcome reports. Overall, our findings indicate that patients with diabetes are at a higher risk of poor hospitalization outcomes, and this is stratified by geographical region. Whilst studies originating from the Far and Middle East reported statistically significant, higher mortality across patients with diabetes, this finding was not the case for the EU, or America world regions (Table 1). Whether healthcare and affordable antidiabetic medication access inequalities or whether inherent non‐modifiable (such as genetic variants) and modifiable parameters (such as obesity) across ethnic groups are responsible for this data variability, should be considered.^3^, ^4^, ^5^, ^6^, ^7^ Furthermore, whilst geographical stratification did not lead to significant differences amongst world regions regarding disease severity in patients with diabetes, the need for ventilation, here defined as either invasive or non‐invasive, was variable across the world. Studies from America, mostly reflecting USA trends, did not indicate higher ventilation requirements in this patient group. Whether this finding reflects overall healthcare system preparedness for catastrophic events, including pandemic emergence is not clear.^173^

**TABLE 1:** Summative results of geographical variation amongst study outcomes

Outcome	America	EU	Far East	Middle East
Mortality [*N* ^total^: 136]	1.42 [1.02.1.97] [*N*:26]	1.47 [1.01, 2.13] [*N*:18]	2.4 [1.97, 2.91] [*N*:77]	1.71 [1.33, 2.19] [*N*:15]
ICU Admission [*N* ^total^: 59]	1.57 [0.6, 4.11] [*N*:13]	1.20 [0.93, 1.55] [*N*:9]	1.94 [1.51, 2.49] [*N*:29]	1.32 [0.82, 2.15] [*N*:8]
Ventilation requirement [*N* ^total^: 83]	0.71[0.42, 1.18] [*N*:14]	1.26 [1.12, 1.41] [*N*:8]	1.61 [1.26, 2.05] [*N*:51]	2.02 [1.32, 3.09] [*N*:10]
Severity (Severe/Critical) [*N* ^total^: 43]	1.36 [1.01, 1.83] [*N*:1]	1.06 [0.39, 2.84] [*N*:1]	2.92 [2.23, 3.84] [*N*:38]	1.98 [1.24, 3.17] [*N*:3]
Discharge [*N* ^total^: 22]	1.20 [0.52, 2.79] [*N*:7]	0.44 [0.25, 0.78] [*N*:3]	0.40 [0.30, 0.53] [*N*:11]	0.76 [0.49, 1.17] [*N*:1]

*Note:*OR 95% CI and number of studies [*N*] employed for the generation of each outcome depicted.

The present work has also highlighted those patients with overall better control of diabetes and on oral glucose‐lowering medications such as metformin, had significantly improved outcomes in terms of mortality. Intriguingly, insulin use has been identified as a risk factor in COVID‐19‐positive, patients with diabetes. As almost the entirety of the patients with diabetes included in the present study, were patients with type 2 diabetes and given that insulin use is the final step in the control of type 2 diabetes, this finding may signify an overall decreased patient physiological reserve or poorer all‐mortality outcomes, as shown in previous studies.^174^ Whilst adjusted hazard ratios for medications amongst patients with diabetes still highlighted an increased risk of death in this patient group, biochemical variables including HbA1C where not consistently reported across studies to enable its inclusion in our adjusted model. Previous work has highlighted that hyperglycaemia in COVID‐19 patients is notable (reviewed in Accili, 2021).^175^ Thus, the literature consensus, in agreement with our findings, supports that good glycaemic control is the best way prevent COVID‐19‐related admissions.^175^ The lack of consistent evidence across studies did not allow for robust comparison of mortality outcomes amongst the patients with type 1 vs. with type 2 diabetes, albeit the clinical need for highlighting hospitalization outcomes in patients with type 1 diabetes. Overall, crude mortality rate for the patients with type 1 diabetes was found to be 18.5% in comparison to 20.1% in the patients with type 2 across the included studies. Whether control of diabetes, in the context of lifestyle and medical interventions rather that diabetes as a diagnosis, is a significant confounder of higher mortality rates across this patient group remains to be clarified and may pose a significant socioeconomic challenge worldwide in the light of the ongoing COVID‐19 pandemic.

### 4.1 Limitations

Our study suffers from the inherent limitations of the included observational studies and the evident lack of RCT studies, which whilst difficult to formulate in the context of a pandemic, would provide further insight in the delineation of diabetes effects upon COVID‐19 hospitalization outcomes. Additionally, whether patients without diabetes as reported per each study were truly representing an unaffected population from diabetes, given that approximately half of diabetes cases remain undiagnosed worldwide, remains obscure and a factor that was not feasible to be controlled in the present study.^177^ Outcomes such as discharge rate which are directly affected by inadequate follow‐up periods may not be truly representative of the final discharge rates across the patients with diabetes, which may require longer hospitalization stays.^76^, ^176^, ^178^ Inconsistent disease severity definitions as well as the consistent lack of BMI as a confounding variable of COVID‐19 mortality across patients with diabetes across studies increased overall reporting bias across the included studies. Lastly, temporal changes in COVID‐19 waves may present significant confounders of mortality reporting across world regions, albeit it should be mentioned that the majority of studies included in the present work have collected patient data during the year of 2020 with specific durations depicted in Table S1, Figure S6.

### 4.2 Strengths and implications for future research

To the best of our knowledge, the present work is the first umbrella meta‐analysis and systematic review, to assess patients with diabetes outcomes regarding COVID‐19 infection whilst accounting for geographical location of outcome reports. We have identified and addressed sources of heterogeneity by geographical and study design subgrouping sensitivity and IVR analysis. This study is the first to highlight major worldwide discrepancies and data variability worldwide in major clinical outcomes. Through this work, we highlight the overall healthcare system preparedness, medication availability and patient ethnicity‐related modifiable and non‐modifiable variables as putative risk factors of worldwide mortality, ICU and ventilation requirements, amongst the patients with diabetes.

## 5 CONCLUSION

Whilst diabetes is undoubtably a poor prognosticator of COVID‐19 infection outcomes, geographical variations across world regions are notable. Whether this finding comes as a result of the variability of healthcare provisions for control and management or patient ethnicity remains to be fully elucidated.

## CONFLICT OF INTEREST

The authors have no conflict of interest to declare.

## AUTHOR CONTRIBUTIONS

**Stavroula Kastora:**Conceptualization (lead); data curation (lead); formal analysis (lead); methodology (supporting); visualization (lead); writing – original draft (lead); writing – review and editing (lead). **Manisha Patel:** Data curation (supporting); methodology (supporting); writing – original draft (supporting). **Ben Carter:** Methodology (lead); writing – review and editing (equal). **Mirela Delibegovic:** Conceptualization (equal); supervision (equal); writing – review and editing (equal). **Phyo Kyaw Myint:** Conceptualization (equal); supervision (equal); writing – review and editing (lead).

## Supporting information


Tables S1‐S2

Figures S1‐S6

## Data Availability

The data used and analysed during the current study are available as online Supplementary Material.
