# Supplementary material for: Impact of diabetes on COVID‐19 mortality and hospital outcomes from a global perspective: An umbrella systematic review and meta‐analysis
Source: Endocrinol Diabetes Metab. 2022 Apr 20;5(3):e00338. doi: 10.1002/edm2.338 (PMC9094465; doi:10.1002/edm2.338)
Supplement: Supplementary file 1 — Tables S1‐S2 [file EDM2-5-e00338-s002.docx]

Impact of Diabetes on COVID-19 mortality and hospital outcomes, a global perspective: An umbrella systematic review and meta-analysis.

Supplementary Figures & Table S. Legends

Table S1. PICO chart

Table S2. Excluded Studies.

Fig. S1. Overall observational study characteristics for COVID-19 positive, patients with diabetes vs. without. Patient age (A), gender pie chart (B), COVID-19 positive patient overall mortality, per DM vs. non-DM population (C), intubation % (D), ICU admission % (E), discharge % (F) in patients with diabetes (DM) vs. without (non-DM). Graphs were designed using GraphPad Prism V 9.0, statistical significance as calculated with unpaired t-test where p-value <0.05 was considered statistically significant.


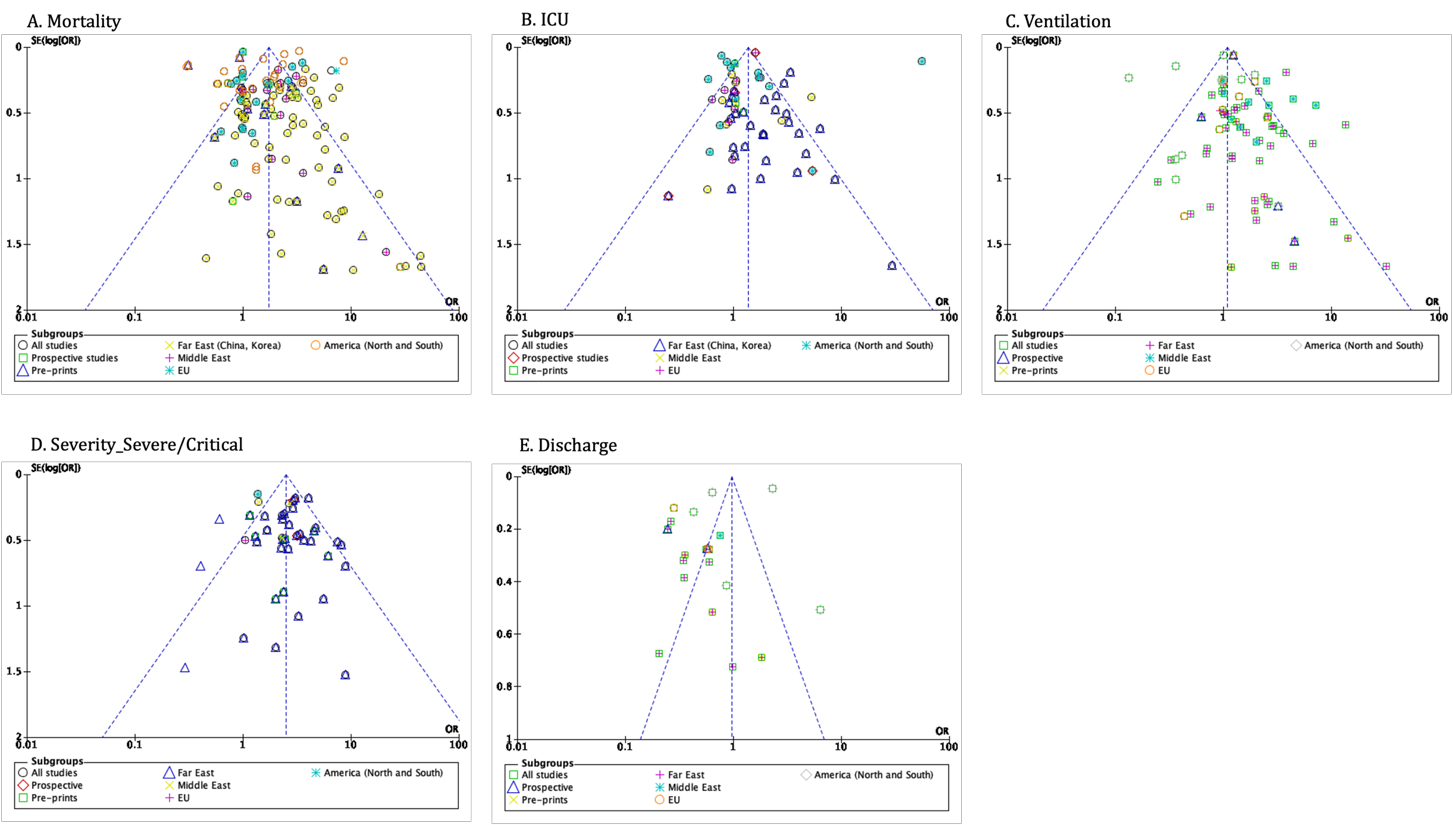


Fig. S2. Publication bias plots for Fig. 2 (A, B), Fig.3 (C, D) and Fig. 4 (E).


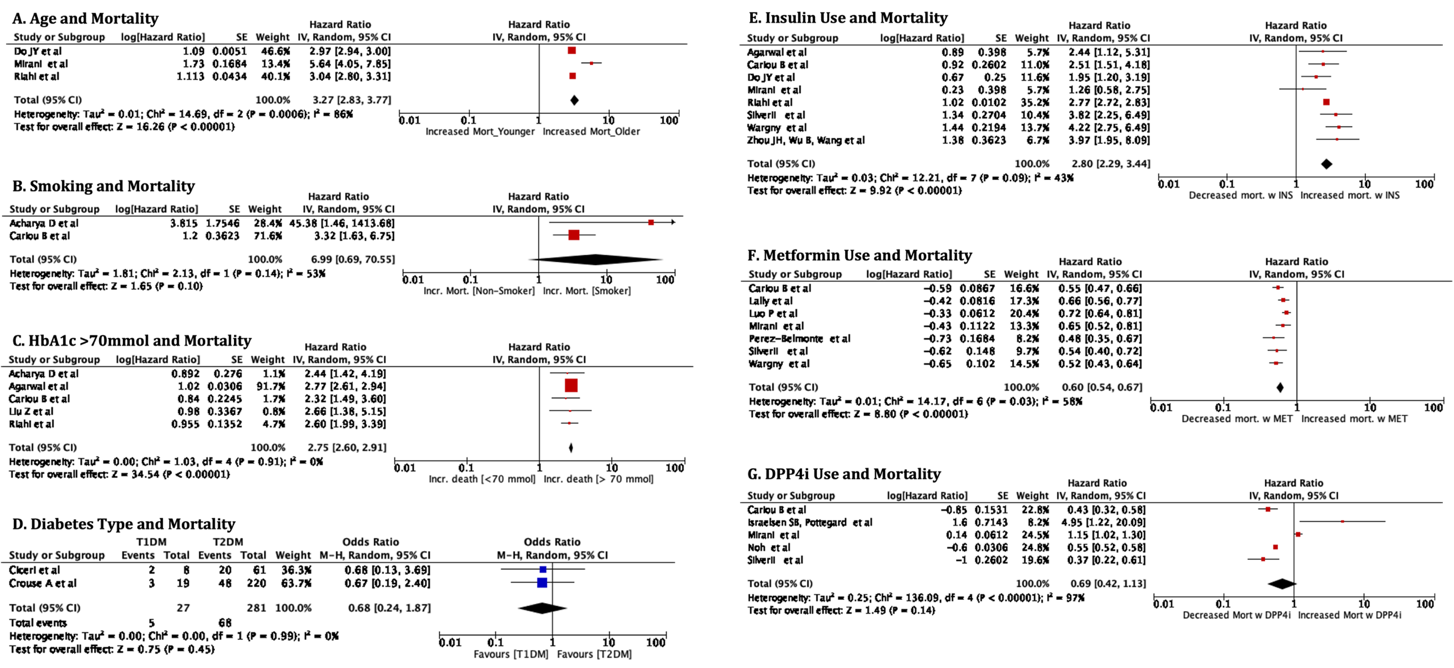
 Fig. S3. Generic Inverse Variance analysis of crude (unadjusted) (A-C, E-G) hazard ratios (95% CI) as reported in each study. (A-D), Age (A), active smoker (B), HbA1c: (Glycated hemoglobin) (C), Haensel-Mantel, random effect, odds ratio associated with decreased mortality between T1DM vs. T2DM patients (D),Insulin use (E), Metformin use (F), DPP4 inhibitor use (G). Image was generated with Review Manager V. 5.4 Cochrane Tool for meta-analysis.


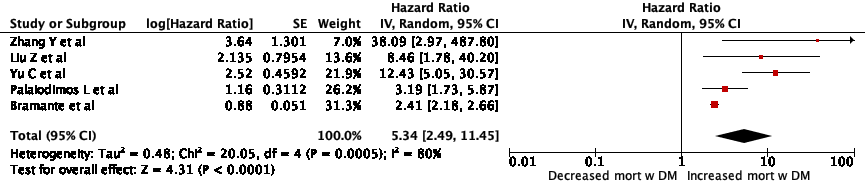


Fig. S4. Generic Inverse Variance analysis of adjusted hazard ratio (95% CI) as reported in each study. HR (95% CI) Model was adjusted for Age; Gender; cardiovascular co-morbidities; Biochemical findings; smoking/alcohol, immunocompromised status), medications. Image was generated with Review Manager V. 5.4 Cochrane Tool for meta-analysis.


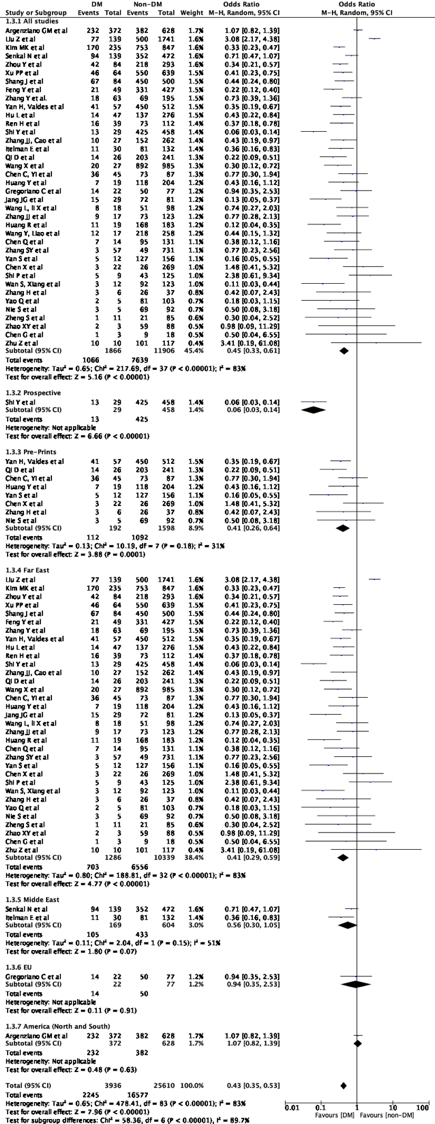


Fig. S5. Uncropped forest plot odds ratio associated with patients presenting with mild condition (all studies depicted). Haensel-Mantel statistical method with odds ratio (random effects) as output only for included observational studies and subgroups as per subgroup title.

Fig. S6. Timeline of mortality [%] amongst total patients (blue) vs. patients with diabetes (pink) between Nov. 2019 till Dec. 2020. Single points and Splines of patient populations. Graphs were designed using GraphPad Prism V 9.0, statistical significance as calculated with unpaired t-test where p-value <0.05 was considered statistically significant.
