# Supplementary material for: Impact of diabetes on COVID‐19 mortality and hospital outcomes from a global perspective: An umbrella systematic review and meta‐analysis
Source: Endocrinol Diabetes Metab. 2022 Apr 20;5(3):e00338. doi: 10.1002/edm2.338 (PMC9094465; doi:10.1002/edm2.338)
Supplement: Supplementary file 2 — Figures S1‐S6 [file EDM2-5-e00338-s001.docx]

Table S1. PICO chart

Table S2. Excluded Studies.
